# Effect of slightly elevated progesterone on hCG trigger day on clinical pregnancy rate in GnRH-ant IVF/ICSI cycles

**DOI:** 10.1186/s12978-022-01371-4

**Published:** 2022-03-14

**Authors:** Jing Zhao, Jie Hao, Bin Xu, Yonggang Wang, Yanping Li

**Affiliations:** Reproductive Medicine Center, Xiangya Hospital, Central South University, 87 Xiangya Road, Changsha, Hunan People’s Republic of China

**Keywords:** IVF, Progesterone, Assisted reproductive technology (ART), GnRH antagonist

## Abstract

**Background:**

It was been agreed that significantly elevated progesterone level on the hCG trigger day have detrimental effect on clinical outcomes in IVF/ICSI cycles. However, few studies explored whether slightly elevated progesterone level also same impact on clinical outcomes.

**Methods:**

We retrospectively studies the effect of slightly elevated progesterone level on outcomes of IVF/ICSI in GnRH-ant cycles. Propensity score matching was used to confounding variables. The women were divided into two groups according to the progesterone level: Group 1: < 1.0 ng/ml; Group 2: 1.0 ng/ml–1.5 ng/ml. Then compare the clinical pregnancy rate (CPR) between the two groups.

**Result:**

A total of 847 IVF/ICSI cycles were included in the present study. The average CPR per transfer cycle was 51.7%. CPR of group 1 was 55.22%, significantly higher than that of group 2 (40.66%, P = 0.013). Progesterone level on the day of hCG injection was further evaluated at threshold increments of 0.1 ng/ml, and the CPR was decreased dramatically once the progesterone level higher than 1.4 ng/ml.

**Conclusion:**

The slight elevation progesterone level on the hCG trigger day may have a negative effect on the clinical pregnancy in GnRH-ant cycles. In the case of progesterone > 1.4 ng/ml on the hCG injection day, freeze-all strategy was recommended.

**Summary:**

The present retrospective study aimed to evaluate the effect of slightly elevated progesterone (1.0 ng/ml ~ 1.5 ng/ml) on outcomes of IVF/ICSI in GnRH-ant cycles. Slightly elevated progesterone level leaded to significant lower clinical pregnancy rate (CPR) that that of group with normal progesterone level (40.66% vs. 55.22%, P = 0.013). The CPR was decreased dramatically once the progesterone level higher than 1.4 ng/ml. So slightly elevated progesterone level on the trigger day may have a negative effect on the clinical pregnancy in GnRH-ant cycles. In the case of progesterone > 1.4 ng/ml on the hCG injection day, freeze-all strategy was recommended.

## Introduction

In the controlled ovarian hyperstimulation (COS) cycles, elevated serum progesterone level is observed in some cases, particularly at the end of stimulation cycle [1]. The incidence of elevated P level was reported as 12.8%–38% in GnRH antagonist cycles [2, 3].

At present, there is no uniform standard for the definition of elevated progesterone levels on hCG trigger day. Most of studies believed that clinical pregnancy rate were reduced when progesterone level > 1.5 ng/ml [4–7], and several studies considered the progesterone > 0.9 ng/ml[8], 1.2 ng/ml[9], even 1.75 ng/ml[10], 2.0 ng/ml[11] as elevated progesterone level. Although the threshold of elevated progesterone level was not consensus, almost all studies found that the significantly elevated progesterone level (P > 1.5 ng/ml) on the hCG trigger day have detrimental effect on clinical outcomes.

What is the relationship between a slight increase in progesterone level of 1.0–1.5 ng/on trigger day and the clinical outcomes? Few studies explored this question. If 1.5 ng/ml > progesterone > 1.0 ng/ml has an adverse effect on the pregnancy outcome, fresh embryo transfer should be cancelled; if there is no adverse effect, the freeze-all strategy would prolong the time of TTB and increase the burden on the patients. Therefore, the purpose of this study is to explore whether slightly elevated progesterone level on the trigger day with 1.0–1.5 ng/ml has an adverse effect on clinical pregnancy in GnRH-ant protocol cycles.

## Material and methods

### Study population and design

The present study was reviewed and approved by the Institutional Review Board and the Ethics Committee of Xiangya Hospital, Changsha, China. The study was based on the Declaration of Helsinki, as revised in 1983. A retrospective, single-center cohort study was performed in Xiangya Hospital, Central South University.

Women undergoing fresh IVF/ICSI-ET with GnRH-ant protocol between January of 2020 and April of 2021 were included in the present study. The inclusion criteria including: (1) 20–40 years old; (2) COS with GnRH-ant protocol; (3) progesterone level was ≤ 1.5 ng/ml on the day of HCG injection. Exclusion criteria including: (1) endometrial polyp; (2) uterine anomaly; (3) donor oocytes or cryopreserved embryos. The basic characteristics were recorded, such as the female age, infertile duration, BMI, COS days, AMH, the E2 and LH level on trigger day, the number of oocytes retrieved, endometrial thickness (EMT).

The present study involved 847 women, and were divided into two groups according to the progesterone level: Group 1: < 1.0 ng/ml (n = 756); Group 2: 1.0 ng/ml–1.5 ng/ml (n = 91). To make the two groups comparable, the propensity scoring matching method was used and women with progesterone < 1.0 ng/ml (n = 364) were matched in a 4:1 ratio to women with progesterone 1.0 ng/ml–1.5 ng/ml (n = 91).

### Ovulation Induction and IVF-ET Procedures

Infertile women used GnRH-ant protocol. Gn (FSH) were given on the third day of menstruation, and the dosage of Gn (FSH) ranged from 150 to 300 IU, according to the women age, AMH, and the number of total antral follicle. The dose was adjusted based on the ovarian response to Gn. When the dominant follicle ≥ 14 mm, E2 ≥ 400 pg/ml, GnRH-ant (Cetrorelix) 0.25 mg was added daily.

Oocytes were triggered with 6000 IU–10000 IU of hCG when the largest two follicles ≥ 18 mm. Oocytes were retrieved 36 h after hCG injection, and followed by conventional IVF/ICSI. Three days after oocyte retrieval, ≤ 2 high-quality embryos were transferred. Vaginal micronized progesterone 0.6 g and oral micronized progesterone 0.2 g were given as luteal phase support, and last for 60 days. The presence of a gestational sac via ultrasound 4–5 weeks after embryo transfer was defined as clinical pregnancy.

### P assessment immunoassay

The electrochemical luminescence was used to test the progesterone levels on the day of hCG injection, with the measured sensitivity and total imprecision of 0.03 ng/ml and < 5%, respectively.

### Statistical analysis

Continuous data were recorded as Mean ± SD, and the student’s *t*-test were selected to conduct the statistics. Categorical data was reported as numbers and the Chi-Square test was used to compare the percentage. The women recruited in the present study were divided into 2 groups according to the level of progesterone on the hCG injection day. SPSS16.0 (SPSS Inc, USA) was implied to carry out the statistical analysis, and the difference was considered to be significance when the *P* < 0.05.

## Results

A total of 847 IVF/ICSI cycles were included in the present study. The average clinical pregnancy rate (PR) per transfer cycle was 51.7%. The average progesterone level on the day of hCG injection was 0.57 ng/ml with a SD 0.28 ng/ml. Other demographic data, such as age, infertile duration, BMI, COS days, AMH, E_2_ level, LH level on the day of hCG injection, the number of oocyte retrieved were summarized in Table 1.

**Table 1:** Characteristics of study group (n = 847)

Characteristics	Mean ± SD
Age (years)	30.79 ± 4.25
Infertility (years)	3.97 ± 3.11
BMI (kg/m^2^)	22.31 ± 3.06
Length of stimulation (days)	9.41 ± 1.76
Total dose of Gn (IU)	1798.65 ± 587.12
Endometrial thickness on hCG day(mm)	10.80 ± 1.97
E_2_ on hCG day (pg/ml)	2393.30 ± 1169.13
LH on hCG day (IU/L)	3.72 ± 2.52
P on hCG day (ng/mL)	0.57 ± 0.28
No. of oocyte retrieved	10.23 ± 4.55
No. of embryos transferred	1.73 ± 0.44

As shown in Table 2, EMT, E_2_ level on the day of hCG injection, number of oocyte retrieved, and clinical pregnancy rate were different between Group 1 and Group 2 (Table 2). Nonetheless, our analysis showed that there was no significant difference in the female age, duration of infertility, BMI, length of stimulation, total dose of Gn, LH on the hCG day between the Group 1 and the Group 2.

**Table 2:** Characteristics of group 1 (P < 1.0) and group 2 (1.0 < P < 1.5)

Characteristics	Group 1 (n = 364)	Group2 (n = 91)	P
Age (years)	31.02 ± 3.78	31.14 ± 4.03	0.512
Infertility (years)	3.99 ± 2.72	4.16 ± 3.74	0.842
BMI (kg/m^2^)	21.87 ± 2.97	21.63 ± 3.11	0.053
Length of stimulation (days)	9.41 ± 1.77	9.41 ± 1.56	0.974
Total dose of Gn(IU)	1813.34 ± 568.15	1842.24 ± 535.47	0.647
Endometrial thickness on HCG day(mm)	10.51 ± 1.78	10.39 ± 2.02	0.042
E_2_ on hCG day(pg/ml)	2674.81 ± 1237.65	3376.93 ± 1197.54	0.025
LH on hCG day (IU/L)	3.87 ± 2.64	4.09 ± 3.46	0.659
No. of oocyte retrieved	10.26 ± 4.51	12.76 ± 4.47	0.047
clinical pregnancy rate % (n/n)	55.22%(201/364)	40.66%(37/91)	0.013

Progesterone level on the day of hCG injection was further evaluated at threshold increments of 0.1 ng/ml to assess its discriminatory ability for clinical pregnancy. Slightly elevated progesterone level leaded to significant lower clinical pregnancy rate (CPR) that that of group with normal progesterone level (40.66% vs. 55.22%, P = 0.013). The CPR was decreased dramatically once the progesterone level higher than 1.4 ng/ml (Table 3).

**Table 3:** clinical outcome according to P level on the day of hCG injection

P level	Cycles (n)	Clinical pregnancy rate (n/%)
0.1 ~ 0.2	33	14 (42.4%)
0.2 ~ 0.3	70	37 (52.9%)
0.3 ~ 0.4	101	55 (54.5%)
0.4 ~ 0.5	118	66 (55.9%)
0.5 ~ 0.6	108	56 (51.9%)
0.6 ~ 0.7	120	65 (54.2%)
0.7 ~ 0.8	86	41 (47.7%)
0.8 ~ 0.9	71	37 (52.1%)
0.9 ~ 1.0	49	30 (61.2%)
1.0 ~ 1.1	37	16 (43.2%)
1.1 ~ 1.2	22	7 (31.8%)
1.2 ~ 1.3	13	7 (53.8%)
1.3 ~ 1.4	13	6 (46.2%)
1.4 ~ 1.5	6	1 (16.7%)
*P*		0.476

## Discussion

So far, many studies have evaluated the effect of elevated progesterone level on the trigger day on the clinical outcomes after IVF/ICSI cycles [11–14]. But few studies analysed whether slight elevation of progesterone level (1.0–1.5 ng/ml) has detrimental effect on the outcomes, especially in GnRH-ant protocol. To the best of our knowledge, the present study assessed the clinical outcomes between slight elevation progesterone level 1.0–1.5 ng/ml with normal progesterone level < 1.0 ng/ml in GnRH-ant protocol cycles for the first time.

In the present study, we found that the clinical pregnancy rate was decreased significantly even with slight elevation progesterone level with 1.0–1.5 ng/ml. In order to assess its discriminatory ability for clinical outcome, the progesterone level was further evaluated at threshold increments of 0.1 ng/ml. The results showed that the clinical pregnancy rate was decreased dramatically when the progesterone level > 1.4 ng/ml.

It was established that serum progesterone elevation on the trigger day has a negative impact on clinical pregnancy outcomes of IVF/ICSI cycles [7, 15, 16], regardless of the development stage of the transferred embryos [10] and trigger with GnRH-a or hCG [11]. The elevation progesterone level ranges from 0.8 to 2.0 ng/ml, according to ovarian response and ovary stimulation protocol [17, 18]. Most studies have suggested that serum progesterone level > 1.5 ng/ml would be consider as premature progesterone elevation and fresh embryo transfer should be cancelled [19].

Although most clinicians think progesterone level < 1.5 ng/ml have no negative effect on outcomes, one study demonstrated that possible deleterious effects may occur when progesterone level < 1.5 ng/ml [10]. Similarly, we found that the clinical pregnancy outcome was poor when the serum progesterone level 1.0 to 1.5 ng/ml in GnRH-ant protocol.

In controlled ovarian stimulation (COS) cycles, multifollicular growth leads to increased ovarian steroidogenic activity and the progesterone elevation might be linked to this mechanism [20, 21]. One study showed that the serum progesterone level on triggering day was closely related to the serum estradiol level, the number of follicles, the number of retrieved oocytes, and ovarian sensitivity. The women’s age, BMI, AFC, and basal FSH, basal E2 were not associated with serum progesterone level on triggering day [22]. Therefore, to avoid elevated progesterone level, milder stimulation approach was recommended to produce fewer oocytes in the following cycles [23].

Increasing number of studies used other indicators, such as progesterone _hCG_/progesterone_basal_ [24], P/E2 [19, 25, 26], progesterone /follicle [19], progesterone /mature oocytes [27], to predict pregnancy outcome. However, the serum elevated progesterone level would induce endometrial transformation, weaken the endometrium receptivity [28], and cause the asynchrony between the endometrium and the embryo [4].

The results found that in the GnRH-ant protocol, a slightly elevated progesterone level will reduce the clinical pregnancy rate. We re-analyzed the GnRH-ant cycles with the progesterone level 1.0–1.5 ng/ml, and found that when the progesterone level was higher than 1.4 ng/ml, the clinical pregnancy rate was significantly reduced. One study found that progesterone level ≤ 0.5 ng/ml on the day of hCG injection hinder live birth rate [6]. Contrast with this study, we did not show the negative effect of lower progesterone level on the clinical pregnancy.

An important strength of the present study was that it is the first study to have compared progesterone < 1.0 ng/ml and progesterone > 1.0 to < 1.5 ng/ml. The main limitation of our study was its retrospective nature. In addition, the present study did not assess the ongoing-pregnancy, live birth and so on.

## Conclusion

The present study suggests the slight elevation progesterone level on the hCG trigger day may have a negative effect on the clinical pregnancy in GnRH-ant cycles. In the case of progesterone > 1.4 ng/ml on the hCG injection day, freeze-all strategy was recommended.

## Data Availability

All data is available in this paper.

## Abbreviations

ART: Assisted reproductive technology
CPR: Clinical pregnancy rate
COS: Controlled ovarian stimulation
